# Bioarchaeological Notes on the Commingled Human Remains Held in the Church of Saint Francis of Paola, Sant’Angelo di Brolo, Sicily, Italy

**DOI:** 10.15388/Amed.2022.29.1.16

**Published:** 2022-07-26

**Authors:** Aurelija Kemežytė, Dario Piombino-Mascali

**Affiliations:** Faculty of Medicine, Vilnius University, Vilnius, Lithuania; Faculty of Medicine, Vilnius University, Vilnius, Lithuania

**Keywords:** commingled remains, anthropology, bioarchaeology, palaeopathology, Sicily

## Abstract

In this study, we examine human skeletal remains from the church of Saint Francis of Paola located in the small town of Sant’Angelo di Brolo, in the Italian region of Sicily. The preserved skeletal remains were temporarily transferred from their permanent resting place in the crypt for a macroscopic examination and evaluation. Various established methods were used to estimate age at death, sex, stature, any evidence of disease(s) as well as the fact that there was a minimum of 15 individuals. The findings were then subdivided into different groups of pathologies and recorded as individual cases. Amongst which, dental issues and cases of trauma were most prevalent. Additional conditions such as joint disease, congenital, metabolic and multifactorial disorders were also identified. The prevalence of dental decay indicates that the group’s diet consisted largely of carbohydrates, and that their oral hygiene was poor. Furthermore, evidence of trauma and poor healing suggested that the town was isolated from the main medical centres of the island. Severe complications of traumas linked with a loss of movement and overall independence, as well as physical pain, must have had a significant impact on the lives of those affected.

## Santrauka.

Šis tyrimas atliktas Sicilijos regiono miestelyje Sant’Angelo di Brolo, Italijoje. Straipsnyje aprašomi žmonių skeletų palaikai, rasti miestelyje įsikūrusioje Šventojo Pranciškaus Pauliečio bažnyčioje. Išlikusios skeletų dalys buvo laikinai perkeltos iš ten esančios kriptos makroskopinei apžiūrai ir įvertinimui. Apskaičiuotas minimalus individų skaičius – 15. Skirtingi standartiniai metodai buvo naudojami vertinti individų amžių mirties metu, lytį, būklę ir ligų požymius. Radiniai suskirstyti pagal skirtingas ligų grupes ir įvertinti kaip individualūs pavienių patologijų atvejai. Odontologiniai sutrikimai ir keletas traumos atvejų išsiskyrė pagal dažnį bendrame radinių kontekste. Taip pat aptikta sąnarių, įgimtų, metabolinių ir daugiafaktorinių patologijų. Karieso atvejų gausa odontologinių ligų grupėje leido įtarti, kad tuo metu mityboje vyravo anglia-vandeniai, o burnos higiena greičiausiai buvo prasta. Dėl su prastu gijimu asocijuotų traumų gausos padaryta prielaida, kad Sant’Angelo di Brolo miestelis buvo įsikūręs atokiau nuo pagrindinių medicinos centrų. Sunkios komplikacijos, susijusios su judėjimo ir savarankiškumo netekimu, taip pat fiziniu skausmu, neabejotinai darė svarbią įtaką paveiktųjų gyvenimui.

## Introduction

**Fig. 1.**
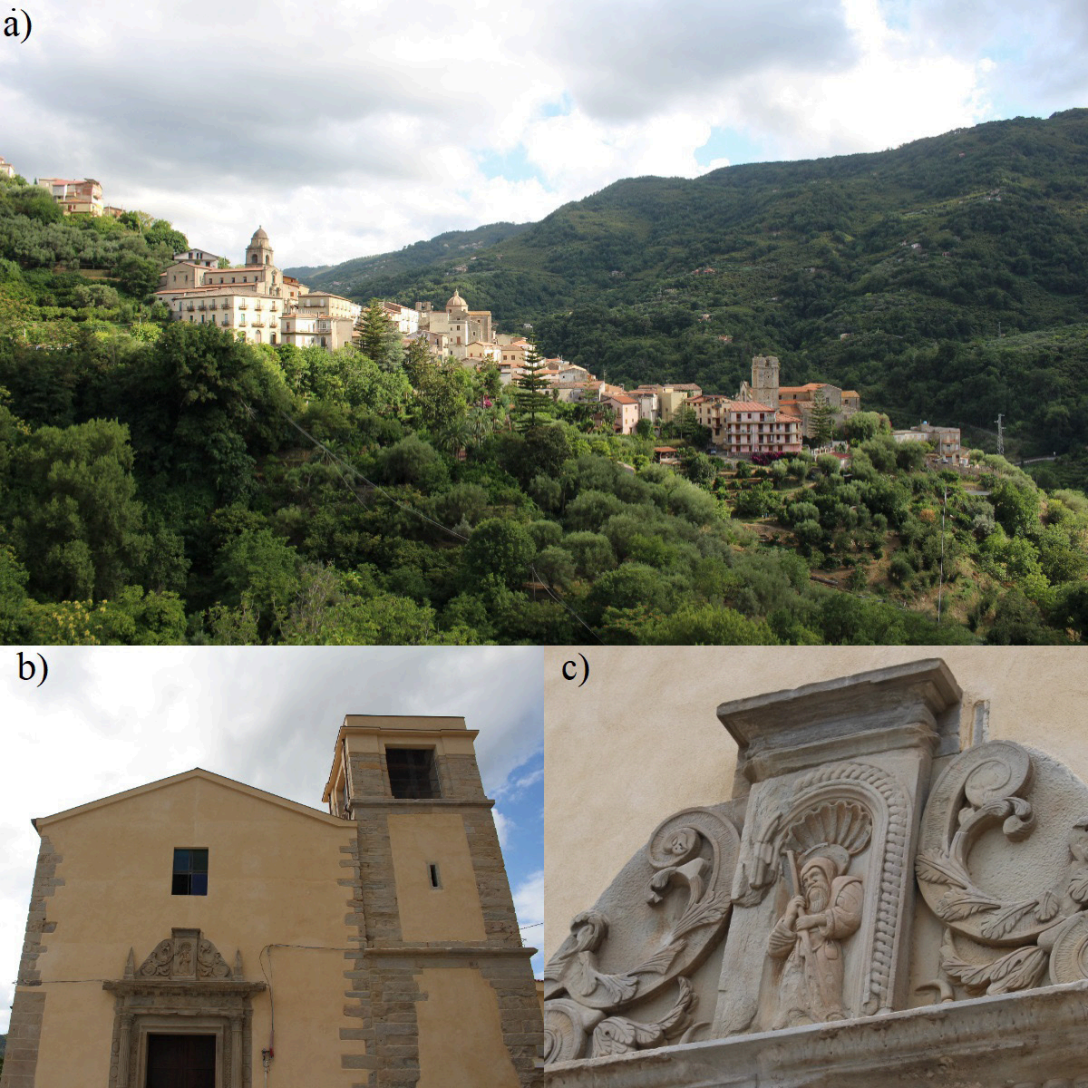
Map of Sicily and location of the site (a); the town of Sant’Angelo di Brolo, Sicily (b); the church of Saint Francis of Paola (c); detail of the main gate of the church (d).

Studies on human remains are of utmost importance for archaeologists as they can provide essential information regarding the circumstances of our ancestors’ lives, behaviour, and medical care. Recently, in the field of bioarchaeology, attention has also been given to extremely damaged and commingled human remains. There are several examples of analyses of fragmentary human bones, such as a study on mortuary practices of an early Roman chamber tomb in Corinthia, Greece [1]. This research was carried out using a highly considered bioarchaeological approach and contextual interpretation following excavation, providing evidence for the role of cremation in local funerary practices [1]. An additional study on commingled remains from rural South Africa focused on bones found in a maize bag and their possible origin. Proven techniques were employed to obtain essential bioarchaeological data, such as: number of skeletal elements present, visual pair matching, articulation, as well as a process of elimination and observation of taphonomic changes [2]. The osteological material investigated in this particular study comes from the town of Sant’Angelo di Brolo, in the province of Messina, Sicily (314 m above sea level), founded in the 11th century by Count Roger I [3]. The remains discussed in this study come from the church of Saint Francis of Paola, which was completed in the 16th century (Fig. 1). The church was a religious building used by the Minims, members of a Roman Catholic religious order of friars. The Minims mostly lived in the convent until the late 19th century, when the premises were confiscated by Italian authorities. In 2019, the aforementioned human remains were found in a crypt during restoration works. In addition, 14 wall niches conceived to facilitate cadaver decomposition were observed, consistent with a mortuary practice common in Sicily, and the general south of Italy [4]. In 2020, the authors of this paper were invited to examine the remains, which were temporarily transferred to a different location for the study.

The initial investigation of these remains suggested that further examination could not be carried out systematically because taphonomic alterations affected most of the bones, leaving them in poor condition. The aim of this study was therefore to present the pathological conditions discovered from the best-preserved remains, emphasise the importance of inspecting commingled remains and speculate about what life was like in Sant’Angelo di Brolo during the time that the church was in use.

## Materials and methods

**Fig. 2.**
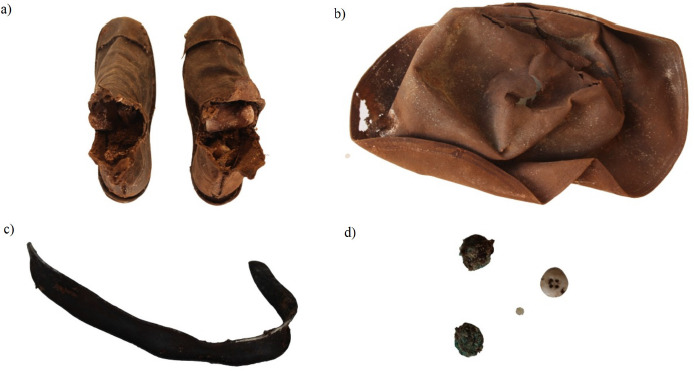
Artefacts associated with the crypt burials including shoes (a); a hat (b); a collar (c); and the buttons (d).

The materials were temporarily moved to the Museum of Angels of Sant’Angelo di Brolo due to the restoration works of the church of Saint Francis of Paola. All bones and bone fragments had been previously organised by niche, location, or deposition, and placed in separate boxes. In the majority of cases, the bone elements were commingled, therefore it was not possible to determine which individual they belonged to. In some of the boxes, fabric fibres, buttons, one collar and several shoes, six of which belonged to children and another two to adults, were also found (Fig. 2). Adult remains were assigned to different age categories, which were tentatively established based on different methods such as: changes in the auricular surface of the ilium, the pubic symphysis, and cranial suture closure [5]. Age-at-death estimation of nonadult remains was carried out through evaluation of tooth development and the length of long bones [6, 7]. Sex was estimated for mature individuals alone by using the morphology of the skull and pelvis [6]. Due to this selective approach and the limited number of well-preserved elements, it was not possible to sort individuals into groups by their biological features. Therefore, we documented the remains by what bones or parts of the bones were present and sorted them by side. Other observations included bone characteristics, size, and articulation patterns [8]. The analysis of pathological conditions and anatomical variations was carried out by describing the bone appearance according to Waldron and Ortner [9, 10]. The most interesting abnormal lesions were illustrated by photographs using a Canon EOS 100D camera. Macroscopic examination, documentation, and literature review were the chosen approaches for this study. Additional methods of analysis, such as radiography, were not available due to the remote location of the town and the rescue nature of the investigation.

## Results

Based on the number of skulls present, it is concluded that a minimum of 15 individuals were observed. Whilst almost all of the subjects were considered to be male or undetermined, we also discovered one female subject. The various types of pathological lesions encountered were individually inspected and organised by type of condition. The findings are listed in Table 1.

**Table 1.** List of diseases presented in this study

No.	Disease	Type of disease
1	Periodontitis	Dental disorder
2	Dental caries	Dental disorder
3	Supernumerary teeth	Dental disorder
4	Periapical abscess	Dental disorder
5	Severe anterior tooth wear	Dental disorder
6	Ankylosing spondylitis	Joint disease
7	Undifferentiated spondyloarthritis	Joint disease
8	Degenerative intervertebral disc disease	Joint disease
9	Eagle’s syndrome	Multifactorial disease
10	Scaphocephaly	Congenital anomaly
11	Cribra orbitalia	Metabolic disorder
12	Distal humeral fracture	Traumatic condition
13	Distal tibial fracture	Traumatic condition
14	Femoral shaft fracture	Traumatic condition
15	Femoral shaft fracture along with hip joint fusion	Traumatic condition

A maxillary bone along with the skull was found, with the sagittal and coronal sutures completely fused. The prominence of the glabella and the well-developed supraorbital ridges suggest that the remains belonged to a male. Evidence of dental wear indicated the remains were of a middle-aged individual. A destructive carious lesion, a process resulting in the demineralization of the dental tissues [9], seen in the form of white and brownish spots on the enamel on the first right molar, was present. The second and the third right molar were lost ante-mortem. On the right first incisor, it was possible to spot two striae of linear enamel hypoplasia, recognised as an increased spacing between the perikymata [11]. The second left molar showed calculus formation and alveolar bone resorption [11].

**Fig. 3.**
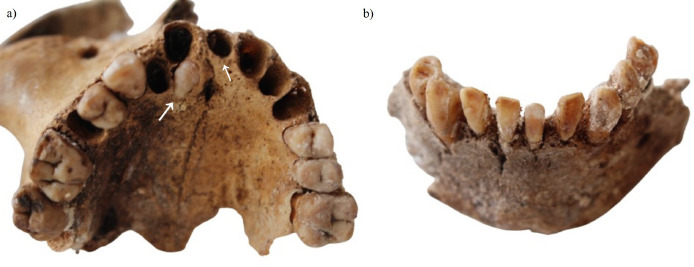
Details of dental pathologies: supernumerary teeth (a); severe anterior teeth wear (b).

Another maxillary bone with some elements of the right zygomatic bone, right canine, first premolar, and two (first and second) molars were found, meanwhile other right teeth were lost postmortem (Fig. 3). On the left side of the maxilla, two premolars and the first molar were observed. The dental wear of this individual suggested that this person was under the age of 50 at the time of his/her death. Two extra teeth, one behind the first right incisor and the other in the area of the first left incisor suggested a supernumerary teeth dental anomaly, defined as an excessive number of teeth in a given individual, commonly associated with genetic disorders [12].

A mandibular bone with left and right side incisors, canines, right first and second premolars, and all left premolars displayed exposed dentin, defined by yellowish and brownish tooth tissue [11]. The incisors showed heavy occlusal wear, which suggested a possibility of parafunction, probably grinding and jaw clenching on the labial surfaces (Fig. 3). Based on the dental wear, it was suggested that this person’s age at death could be over 50 years old. Alveolar bone loss from the cementoenamel junction, as well as the presence of calculus, defined as calcified plaque and colonies of microorganisms [9], indirectly indicated periodontal disease. The upper jaw of this individual was not identified; consequently, the ultimate cause of the anterior tooth wear could not be established.

The last bone with dental pathology was a mandible with the right second incisor, canine, and first and second premolar, as well as the left canine and two premolars present. While the second incisor was lost post-mortem, the remaining teeth were lost during life. Based on the dental wear, this was an adult individual. The anterior alveolar wall of the right first premolar was destroyed, leaving a cavity over 3 mm wide exposed, which suggested the presence of an abscess [11].

**Fig. 4.**
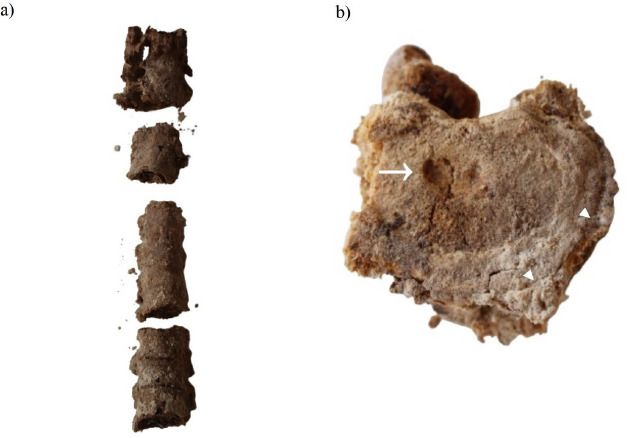
Two examples of spinal diseases including ankylosing spondylitis (a); and Schmorl’s node defect (b).

Additionally, spinal fusion of the cervical to lumbar trait was found (Fig. 4). The poor and fragile condition complicated the inspection, but a noticeable complete ankylosis of the entire segment with no skip lesions and undulating contour indicated the presence of ankylosing spondylitis, a disorder that induces joints to fuse together and acquire a bamboo-like appearance [10].

Two second and third cervical vertebrae were also recognised. The axis appearance indicated the presence of bony spurs around the apex, with the third vertebra demonstrating marginal osteophyte formation around the body and superior articular processes, representing typical signs of undifferentiated spondyloarthritis, an inflammation of the spinal joints that generally involves the soft tissue [9].

On a separate lumbar vertebra, osteophyte formation around the body margins was noticed and suggested the presence of degenerative intervertebral disc disease. Additionally, a Schmorl’s node defect (Fig. 4) herniating the superior surface of the vertebral body was observed, indicating that the individual experienced stress on his lower back [9].

**Fig. 5.**
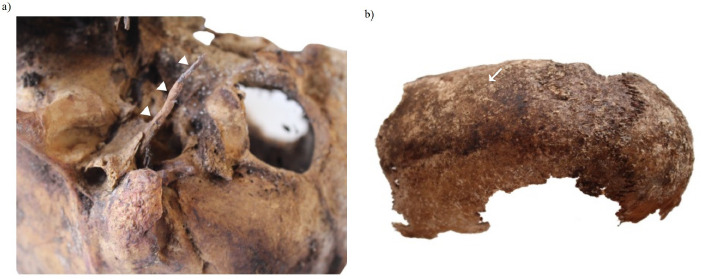
Detail of a case of Eagle’s syndrome (a); detail of a case of scaphocephaly (b).

A case of Eagle’s syndrome was identified based on the appearance of a well-preserved skull, which demonstrated an elongated right styloid process estimated to be 3.5 cm in length (Fig. 5). The left process, however, was broken. The complete closure of the lateral-anterior cranial sutures suggested that this individual was over 50 years old. A large nuchal crest with a well-defined bony ledge and large mastoid process along with a clear prominence of the glabella confirmed the male skull. New bone formation on the right condylar process confirmed the presence of osteoarthritis.

A juvenile individual skull with premature closure of the sagittal suture was also discovered (Fig. 5). The remaining fragments, the frontal and parietal bones, were fused together. The presence of a completely fused sagittal suture clearly suggested a manifestation of a scaphocephaly disorder, or a narrow and elongated head, developed in this manner because of an early fusion of the aforementioned suture [10].

**Fig. 6.**
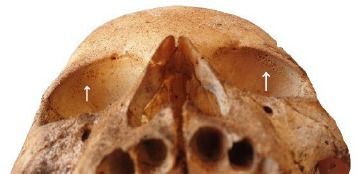
Detail of cribra orbitalia seen in the orbits of one subject.

The presence of a metabolic disorder was identified on an immature skull aged around six years, whereby both orbits were marked by pitting on the superior wall (Fig. 6), indicating the occurrence of cribra orbitalia (a manifestation of several pathological conditions including anaemia) [9].

**Fig. 7.**
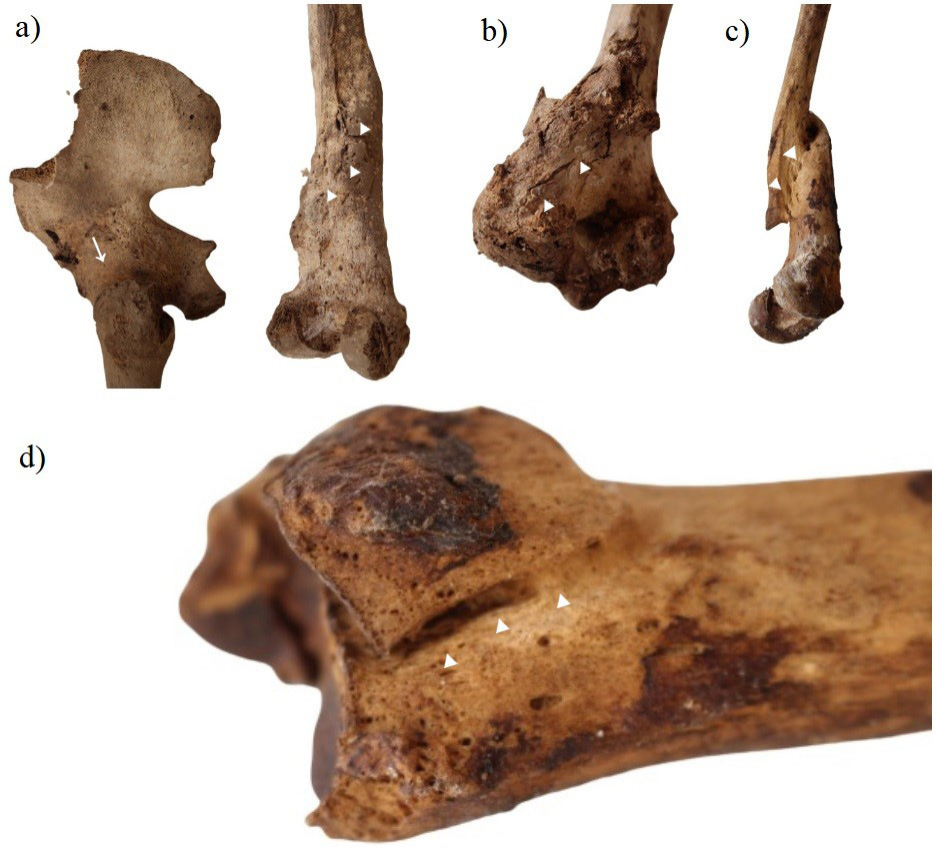
Details of trauma cases including a left femoral bone fused with the acetabulum and new bone formation of the lower third of the shaft (a); a left humerus with a callus formation on the distal end (b); a right femoral bone with significant shortening and callus formation of the lower third of the shaft (c); and a right tibia with new bone formation on the distal epiphysis (d).

We also discovered some signs of trauma on the upper and lower limb bones (Fig. 7). Initially, we identified a lesion starting from the medial supracondylar ridge of the left humerus and continuing to enter the olecranon fossa (Fig. 7). This lesion exhibited signs of healing, defined as a callus formation on the distal end [9]. Based on the complete fusion of the humeral head, this belonged to an adult individual.

A right tibia (which based on the epiphyseal fusion of the tibia belonged to an adult individual) was characterised by new bone formation on the distal epiphysis (Fig. 7). The lesion within the callus formation crossed the medial malleolus. On the posterior part of the distal epiphysis, a mal-united fragment of the lateral malleolus was also visible.

Additionally, a right femur bone with significant shortening and callus formation of the lower third of the shaft was found (Fig. 7). Complete epiphyseal fusion of the femoral head led us to believe that this was an adult individual. The oblique lesion starting from the lower third of the shaft joining a mal-united proximal part suggested it was an oblique fracture. The shaft of the femoral bone was bent medially due to mal-union.

Furthermore, a left femoral bone fused with the acetabulum showing new bone formation of the lower third of the shaft was observed (Fig. 7). The narrow appearance of the sciatic notch clearly indicated that the individual was a male. A dense irregular auricular surface suggested an age of over 50 years old. The complete fusion around the hip region was marked by pitting on the acetabular rim. A huge cloaca with pitting around the entrance entering the obturator groove region of the hip was noted. The distal third fracture of the femoral bone corresponded to infectious changes described before as pitting on the fused parts of the shaft with callus formation. Most of these were well-expressed on the posterior surface of the femoral bone, starting from the middle part of the shaft to the medial and lateral condyles.

We also encountered two lesions that represented nonspecific infectious diseases: sinusitis and periostitis. On the wall of the fragment of the maxillary bone sinus, it was possible to note new bone formation, confirming the presence of chronic sinusitis, a maxillary sinus inflammation commonly caused by bacterial pathogens and producing a blockage of the maxillary bone drainage channel located on the medial wall [9].

Additionally, a well-preserved right tibial bone was found with areas of periosteal new bone formation between the anterior and lateral margins of the middle shaft. This type of bone reaction is most commonly identified as a sign of periostitis. Based on the epiphyseal fusion this tibia belonged to an adult.

## Discussion and concluding remarks

Studying commingled, disturbed, and damaged burials should not be avoided due to challenges that may arise in analysis. These assemblages could provide useful information for anthropologists, historians, and other specialists interested in our ancestors, including their features, social behaviour, and general medical status [13].

The dental pathologies we found mostly convey dietary and masticatory patterns among the inspected individuals. The presence of caries, as well as the periapical abscess formation, which is known to be a consequence of the former [9], suggested that the diet among the individuals who lived in Sant’Angelo di Brolo could have been high in carbohydrates, playing a key role in the formation of carious lesions [14]. Also, poor oral hygiene probably contributed to the observation of caries, as in Europe this was neglected until the beginning of the 19th century [15]. A case of enamel hypoplasia may possibly suggest that the individual concerned had sustained systemic stress, although localised trauma should not be ruled out. Trauma or periapical infection can produce defects on the labial surface of the permanent incisor [16]. Masticatory patterns of the individual with severe anterior tooth wear on their mandibular teeth likely caused this damage, as dietary habits alone could not explain its severity and dominance. Current literature suggests that the intensive use of teeth as tools, such as holding materials in the mouth while sewing, making baskets, or tightening lines, could strongly contribute to this type of pathology [17]. When it comes to the supernumerary teeth, this is usually associated with congenital genetic disorders and syndromes, which frequently results in complications, including but not limited to dental impaction, delayed eruption, ectopic eruption of an adjacent tooth, and dental overcrowding [12]. Nowadays, ectopic teeth are typically removed via surgery [12], but it is evident that this individual did not receive or seek medical help, as the teeth were either present or lost post-mortem.

In bioarchaeology, the most frequently observed bone conditions are diseases of the joints, which can also provide information about the individuals’ quality of life. For example, a case of ankylosing spondylitis, one of the sero-negative spondyloarthritis group of diseases, must have harmed this individual’s well-being [9]. The development of a final stage disease must have resulted in suffering from clinical symptoms, such as lower back pain, limited chest expansion, and immobility. Furthermore, the presence of osteoarthritis affecting the odontoid process of the second cervical vertebra is uncommon, but it is not discussed a great deal in clinical reports, probably because it may be asymptomatic [18].

Regarding Eagle’s syndrome and scaphocephaly, it is difficult to say what impact on the quality of life they had, because those diseases are mostly associated with an unknown, genetic, or congenital aetiology. Eagle’s syndrome, or a styloid process longer than 30 mm can be asymptomatic, but in some cases it can cause symptoms such as the compression of the cranial and cervical sympathetic nerve, unilateral persistent neck pain, as well as dysphagia [19]. The congenital disorder, known as scaphocephaly, normally has no complications [20].

Metabolic disorders like cribra orbitalia are frequently studied in bioarchaeology [9, 10]. Even though this condition is commonly associated with different types of anaemia, nowadays it is known that a connection between respiratory tract infections and cribra orbitalia exists, which suggests that it could be one of the explanations for the relatively large sample of juvenile skeletal elements found in the crypt of the church [21]. In fact, it is known that respiratory tract infections (diphtheria, pulmonary tuberculosis, scarlet fever) were a serious problem during childhood until the late 19th century [22].

Regarding the periosteal bone reaction seen in the middle part of the tibia, at this anatomical location it commonly results from repeated minor trauma [9]. As with sinusitis, there is nothing specific about the infective agents, and the complications are less frequent [9].

As far as the injuries are concerned, the oblique fracture of the right femur of an adult individual with significant shortening, mal-union, and callus formation may have resulted from injury with a combined/angulated force [23]. A mal-union is the result of nonmaintained reduction of an injury and the fragments left to heal angulated and shortened [23]. Another mechanism that promotes the formation of this deformity is the activity of the psoas and abductor muscles, whereby, the proximal segment is pulled into flexion and external rotation while the distal fragment is drawn into varus position by the adductors, shortening is caused by the extensor muscle’s forces [24]. Shortening of a lower limb as a complication of a fracture is tolerable if it is up to 20 mm; however, a greater loss in length can produce lateral and rotational spinal deviation and backache from pelvis tilting [23]. It could be speculated that these complications affected our described individual, as the shortening of the femur was certainly exceeding that measure. Traditionally, activities like agriculture, forestry, carrying water, and farming have high fracture risks [23]; however, it is unlikely that individuals from this crypt were heavy workers, because crypts of this kind were typically used as final resting places for saints, priests, and others deemed worthy of entombment within a church [13]. The most plausible explanation is that the individuals from the Sicilian town of Sant’Angelo di Brolo did not receive sufficient medical care, most likely due to its remote location, far from the better developed cities on the island.

The right tibia of an adult individual represented a bimalleolar fracture with evidence of healing on the medial malleolus and dislocation with mal-union of the lateral malleolus on the posterior part of the tibia. This type of injury to both sides of the ankle joint results in rupture of the anterior syndesmotic ligament and has an unstable ankle mortise, which requires surgical treatment [25]. This causes foot supination and external rotation. Therefore, in our case, the most probable mechanism would have been inversion and hyper-dorsiflexion trauma with injured anterior talofibular ligament, which is classified as Weber type B fracture [26]. If not repaired surgically, complications may include dislocation, limited movement, compartment syndrome, or even complex regional pain syndrome [27]. It is worth mentioning that one-year mortality after operative intervention accounts for 12% of deaths among individuals older than 65 years with this type of injury; however, we can only speculate how the consequences could have influenced the time of death of this individual [28].

Another rare fracture of the distal part of the left humerus correspondingly represents the same pattern of activity-induced trauma. With reference to AO/OTA classification, it is an intra-articular single column (type B) fracture [29] based on the visible fracture line and the callus formation on the lateral part of the distal humerus. The incidence of humeral fractures in the current century (calculated by the General Register of the Scottish Office) comprises 2% of fractures in adults [30]. At present, it is believed that in patients under 50 years of age the predominant mechanism of injury is high-energy trauma such as falls from heights, while in the other group (over 50 years) simple falls are a frequent way to sustain a humeral fracture [31]. Currently, the treatment for this type of fracture is surgery, and nonoperative treatment is associated with poor outcomes [32], such as loss of movement affecting daily living activities and reduced self-sufficiency [33].

The last trauma case, arguably the most interesting and complex one, is represented by the left femur that was completely fused within a hip joint. Because of well-spread osteomyelitis within this bone, we believe that an oblique fracture of the distal part of the femur was the consequence of an untreated infection [9]. Due to local spread from a contiguous contaminated source of infection, osteomyelitis often develops following trauma [34]. Based on the processes that occurred in the bone (fusion, callus formation, cloaca), it can be speculated that it was a chronic infection that evolved over months or even years [34]. Despite haematogenous osteomyelitis being rare in adults [9], in our case this could be a result of the distal fracture, which may be explained by the possible fracture of the femoral head entering the joint space and the great cloaca near the obturator groove, possibly representing the primary source of infection. However, because imaging methods were not used in this study, it is difficult to confirm this hypothesis. Contemporary antibiotic prophylaxis is successfully used to prevent these infections [34], but this treatment was not available until the discovery of effective antibiotics in the 20th century [9]. On the contrary, we cannot exclude other causes of hip fusion, such as tuberculosis, which can also result in a pathological fracture. Therefore, further examinations should be carried out to clarify the exact cause of these changes.

In conclusion, although highly damaged and in poor condition, the commingled remains investigated at Sant’Angelo di Brolo revealed the presence of interesting abnormalities, useful for both educational and historic purposes, some of which are not commonly encountered in day-to-day medical practices. Long-term storage in the restored crypt allows for future radiological examination to shed additional light on the overall pathological picture of those remains.

## Limitations to the study

As indicated previously, this study was affected by a number of limitations including overall preservation of the remains and the logistics associated with carrying out the research on site. Furthermore, additional analytical methods such as radiography would have enabled the authors to interpret the results more precisely. Lastly, contextual historic data on the individuals buried in the crypt could not be obtained at this time as the church archive was not accessible.
